# Citalopram-induced photopigmentation

**DOI:** 10.1016/j.jdcr.2026.06.059

**Published:** 2026-07-03

**Authors:** Lily Kaufman, Sonali Batta, Nada Mohamed, Katherine Fiala

**Affiliations:** a The Ohio State University College of Medicine, Columbus, Ohio; b Department of Dermatology, Baylor Scott & White Health, Temple, Texas; c Department of Dermatopathology, Baylor Scott & White Health, Temple, Texas

**Keywords:** drug-induced photopigmentation, drug-induced pigmentation, hydroquinone, kojic acid

## Introduction

Drug-induced pigmentation (DIP) accounts for approximately 20% of acquired hyperpigmentation^1^ and arises through either increased melanin production, pigment deposition, or drug-melanin complex formation.^2^ A subset of these cases presents as drug-induced photopigmentation (DIPP), in which discoloration occurs predominantly in sun-exposed areas due to photosensitizing properties of the medication. DIPP has been most commonly associated with agents such as tetracyclines, antimalarials, amiodarone, phenothiazines, and certain chemotherapeutic or antiretroviral drugs. The clinical appearance onset, and distribution each vary depending on the drug and mechanism involved. We present a case of citalopram-induced blue-gray photopigmentation affecting the face and forearms.

## Case report

**Fig 1:**
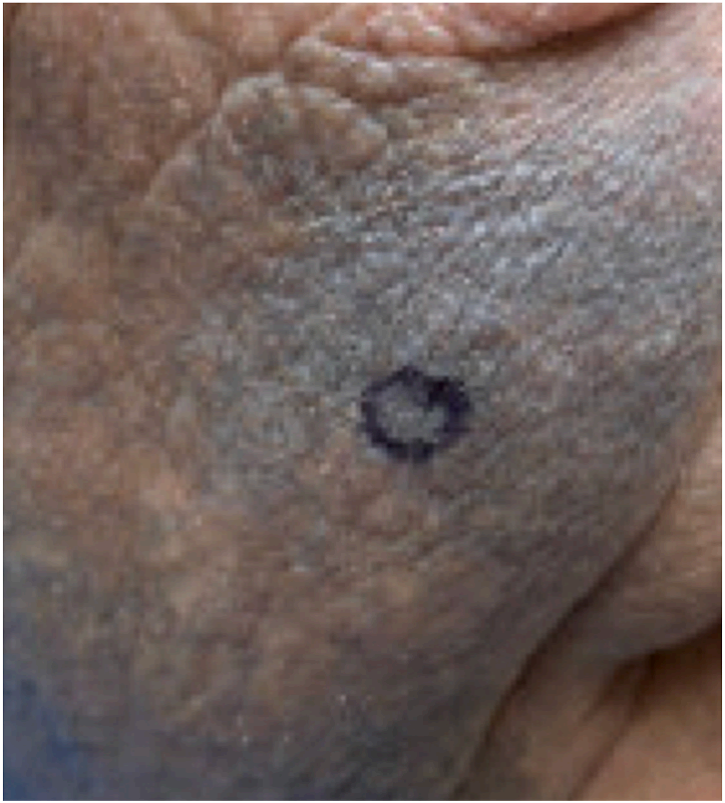
Blue-gray dyspigmentation of the right facial cheek.

**Fig 2:**
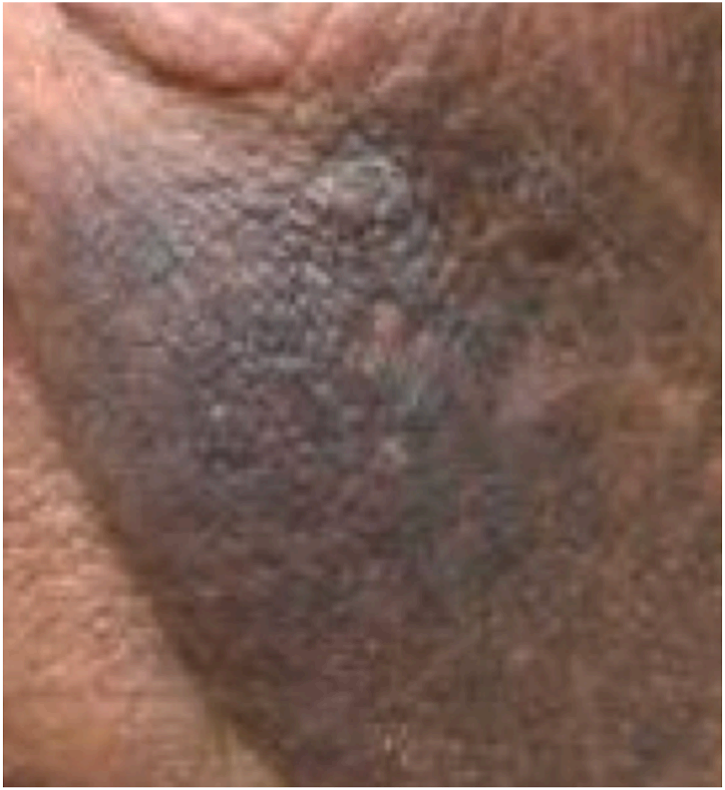
Blue-gray dyspigmentation of the left facial cheek.

A 63-year-old White male presented to our dermatology clinic with blue-gray photopigmentation of sun-exposed areas of approximately 5 years’ duration. He began taking citalopram 5 years prior for generalized anxiety disorder. Other medications included clonidine, pravastatin, sildenafil, and trazodone, which were all started greater than 5 years prior. Testosterone began within the past year. He denied prior or current use of amiodarone, minocycline, phenothiazines, antimalarials, imipramine, chemotherapeutic agents, prolonged (>14 day) courses of doxycycline, herbal supplements, kratom, or known exposure to heavy metals. Physical examination showed blue-gray photopigmentation of the facial cheeks (Figs 1 and 2) and bilateral forearms extending from the extensor elbows to the proximal interphalangeal joints. No mucosal involvement was noted on examination.

**Fig 3:**
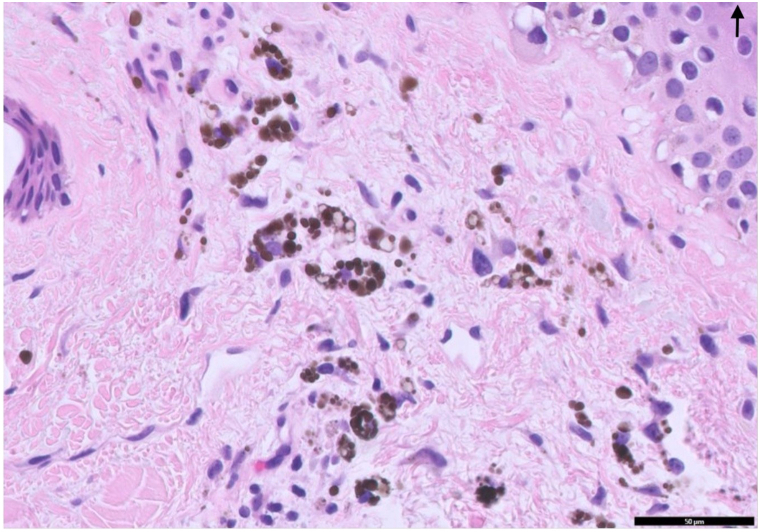
Prominent pigment deposition within the papillary dermis (H&E, 40×).

A punch biopsy performed of the patient’s right cheek demonstrated prominent pigment deposition within the papillary dermis (Fig 3). No melanocytic proliferation or deposition of silver pigment was identified. Additional staining was Fontana-Masson positive and iron negative. Pathology results were consistent with DIP.

Aside from pigmentary changes, the patient had no associated symptoms. Citalopram was discontinued, and the patient began a compounded topical hydroquinone 12% and kojic acid 6% cream with diligent sun protection. Laser therapy was discussed as a potential treatment option, but was deferred due to cost.

At his 3-month follow-up visit, the patient noted only mild improvement with the compounded cream. Oral tranexamic acid was not recommended due to the patient’s elevated thrombotic risk. Given limited response to topical therapy, we recommended laser treatment at an external dermatology facility. The patient has not yet pursued this.

## Discussion

DIPP has been reported in the literature with various medications, including antibiotics, antimalarials, antipsychotics, antiretrovirals, heavy metals, prostaglandin analogues, and chemotherapeutic agents. Onset, duration, appearance, and distribution of DIPP varies by causative agent, making a detailed medication history and timeline essential for identifying the culprit medication.^1^ DIPP occurs through 3 main mechanisms. First, some drugs stimulate melanocytic activity or local inflammation, producing excess melanin that accumulates in the dermis either freely or within macrophages. Second, drug-melanin complexes may form, and ultraviolet light exposure can impair their clearance, leading to photo-distributed pigmentation. Third, certain medications deposit directly in the dermis as granular material, either extracellularly or within macrophages, contributing to photopigmentation. Other pathways include the drug-driven synthesis of pigments such as lipofuchsin and deposition of iron following drug-induced vascular damage and red blood cell leakage.^2^

DIPP has been reported in patients taking tricyclic antidepressants such as amitriptyline and imipramine, described as slate-gray or blue-gray pigmentary changes in a photodistribution.^3^ Less frequently, dyspigmentation associated with SSRIs has been reported. To date, only one case report has described diffuse photopigmentation following initiation of citalopram,^4^ and development of citalopram-induced oral melanotic macules has also been observed.^5^ Although causality cannot be definitively established, citalopram was considered the most likely culprit for our patient given the temporal association with symptom onset, compatible histopathologic findings, photodistributed pattern, and exclusion of other commonly implicated medications and exposures.

Management of DIPP may be challenging, as resolution may be slow and incomplete even after discontinuation of the offending medication. Topical depigmenting agents such as hydroquinone, azelaic acid, kojic acid, tranexamic acid, and retinoids may be used to provide gradual improvement by reducing melanin synthesis and enhancing epidermal turnover.^6^ However, their efficacy in DIP may be limited when pigment deposition occurs in the dermis, as these agents predominantly target epidermal melanogenesis.^6^, ^7^, ^8^ Chemical peels, particularly those using salicylic or glycolic acid, can also be considered but require caution in patients with photodamaged or sensitive skin, as they may precipitate postinflammatory hyperpigmentation.^6^^,^^7^ Laser therapy, including Q-switched ruby, alexandrite, and Nd:YAG lasers, has demonstrated efficacy in targeting dermal pigment, although outcomes vary depending on depth of pigment deposition and skin phototype. Lasers are often considered when topical therapy is insufficient, and recurrence or postinflammatory pigmentation are possible risks. Additionally, cost and access may be limiting factors,^1^^,^^9^ as in the case of our patient, thus restricting our assessment of potential treatment response. In particular, the Q-switched alexandrite laser (755 nm) has shown benefit in psychotropic and other drug-induced hyperpigmentation, including imipramine- and minocycline-associated pigmentation. Reported treatment parameters in the literature typically include fluences of 3-8 J/cm^2^, 3-4 mm spot sizes, and multiple treatment sessions spaced several weeks apart.^10^, ^11^, ^12^ In all cases, strict photoprotection is a critical part of management, as UV exposure can exacerbate pigmentation and undermine treatment progress.^6^^,^^7^ Although this report describes a single case, recognition of rare medication-associated pigmentary reactions remains important for dermatologists evaluating new-onset photodistributed dyspigmentation. In summary, citalopram is a rare but potential trigger of DIPP, and treatment options vary in cost and efficacy, requiring careful consideration to balance patient goals and resources.

## Conflicts of interest

None disclosed.
